# Gastric xanthelasma is a warning sign for *Helicobacter pylori* infection, atrophic gastritis, and intestinal metaplasia

**DOI:** 10.3389/fmed.2023.1252346

**Published:** 2023-09-01

**Authors:** Lina Feng, Mingyu Zhang, Jialun Guan, Yu Zhang, Yujie Huang, Ruonan Dong, Kai Zhao, Suhong Xia, Fang Xiao, Jiazhi Liao

**Affiliations:** Department of Gastroenterology, Tongji Hospital of Tongji Medical College, Huazhong University of Science and Technology, Wuhan, Hubei, China

**Keywords:** xanthelasma, *Helicobacter pylori*, intestinal metaplasia, dysplasia, gastric cancer, gastric atrophy

## Abstract

**Background:**

Contradictory evidence suggested gastric xanthelasma (GX) was associated with some upper gastrointestinal (GI) diseases. Additionally, no research has been performed on the relationship between esophageal/duodenal xanthelasma and upper GI diseases.

**Methods:**

Individuals who underwent esophagogastroduodenoscopy at Tongji Hospital, Tongji Medical College, participated in this retrospective study. This study evaluated whether the risk of GX or esophageal/duodenal xanthelasma was influenced by the following gastroesophageal diseases: superficial gastritis, gastric polyp, bile reflux, peptic ulcer, reflux esophagitis, Barrett’s esophagus, esophageal cancer, atrophic gastritis (AG), intestinal metaplasia (IM), dysplasia, gastric cancer, and *Helicobacter pylori* (*H. pylori*) infection. Furthermore, subgroup analysis was conducted to establish the relationship between the number of GX and upper GI diseases.

**Results:**

Of the 69,071 subjects reviewed, 1,220 (1.77%) had GX, and 54 (0.08%) had esophageal/duodenal xanthelasma. There was no difference in the prevalence of upper GI diseases between patients with and without esophageal/duodenal xanthelasma. Nevertheless, compared with non-xanthelasma patients, GX patients had a greater proportion of AG, IM, dysplasia, gastric cancer, and *H. pylori* infection and a lower incidence of superficial gastritis (*p* < 0.05). The multivariate logistic regression analysis indicated AG (OR = 1.83, 95%CI: 1.56–2.16), IM (OR = 2.42, 95%CI: 2.41–2.85), and *H. pylori* infection (OR = 1.32, 95%CI: 1.17–1.50) were independent risk factors for GX. In addition, patients with multiple GXs had a higher rate of AG and IM than those with single GX.

**Conclusion:**

Esophageal/duodenal xanthelasma may not be associated with upper GI diseases, and further research is needed to support this hypothesis. Notably, GX, especially multiple GXs, may be a more easily detected warning sign of AG, IM, or *H. pylori* infection.

## Introduction

Xanthelasma is a rare benign lesion encountered during upper gastrointestinal (GI) endoscopy examination. As early as 1887, Orth (1) first recorded this entity. Subsequently, some terms such as “xanthoma” and “lipid island” have been successively used to describe this disease (2, 3). Now, the term “xanthelasma” is widely accepted and used (4, 5). It appears as a yellow-white nodule or plaque under endoscopy, with a size ranging from 1 to 10 mm (6). The most common site is the stomach which also occurs in the esophagus and duodenum. The reported prevalence of gastric xanthelasma (GX) ranges from 0.018 to 7.7% (7–13).

As is well known, *Helicobacter pylori (H. pylori)* is a microaerophilic, gram-negative bacterium commonly found in the stomach (14). The chronic inflammation of gastric mucosa induced by its infection is closely related to the progression of the gastritis-atrophy-metaplasia-dysplasia-cancer sequence (15). In recent decades, limited research has explored the correlation between GX and various factors, such as *H. pylori* infection, atrophic gastritis (AG), intestinal metaplasia (IM), and gastric cancer (8, 13, 16–19). However, most studies have a small sample size, and the results are still inconclusive. In addition, no study has investigated the clinical significance of esophageal/duodenal xanthelasma.

Consequently, we performed a large retrospective study investigating the relationship between xanthelasma (including gastric and esophageal/duodenal) and upper GI endoscopic or histopathological features. Moreover, we also evaluated whether AG or IM plays some role in the relationship between *H. pylori* infection and GX.

## Materials and methods

### Patients

**Figure 1:**
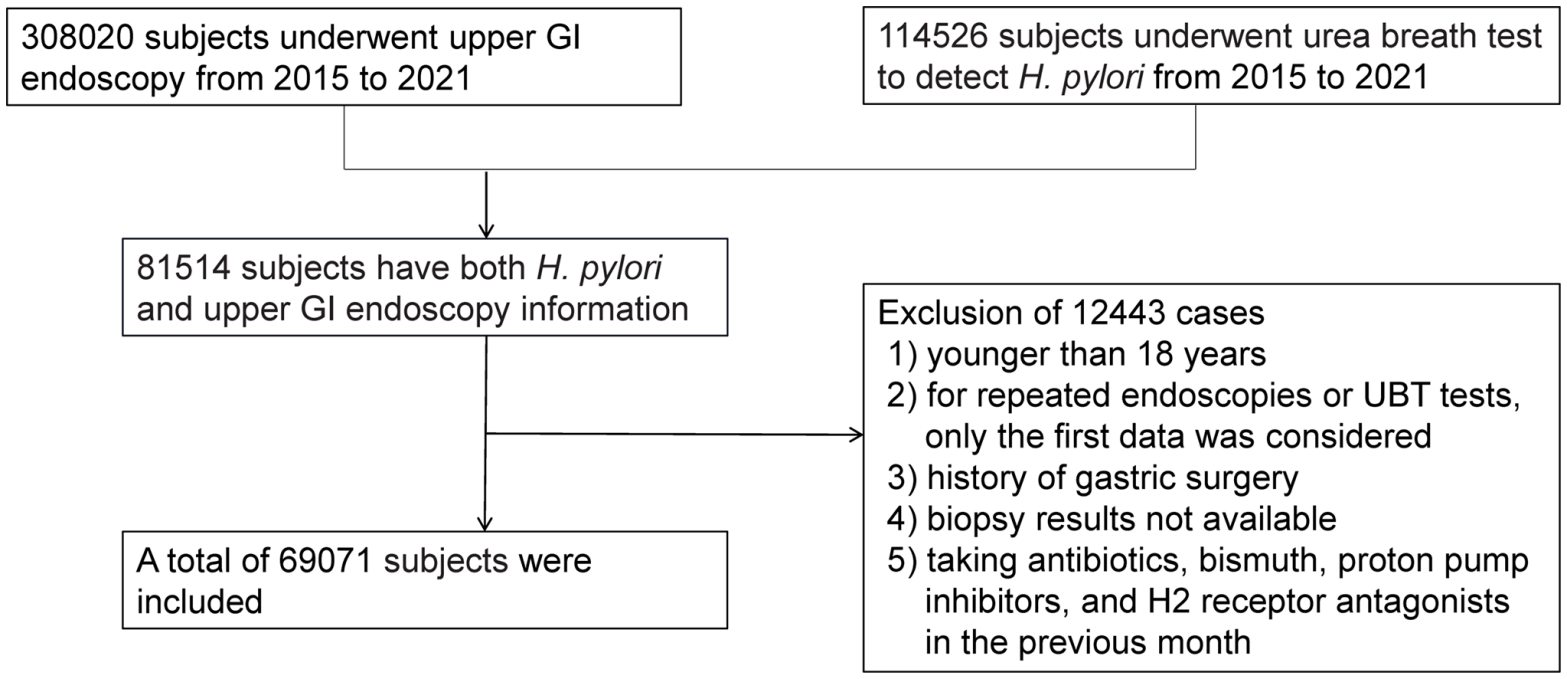
Patient flow chart.

We performed an observational study of patients who underwent upper GI endoscopy at our institution between January 2015 and November 2021. During this period, 308,020 subjects underwent gastroscopy, and 114,526 subjects underwent a urea breath test (UBT) to detect *H. pylori*. Eighty-one thousand five hundred fourteen subjects who had both *H. pylori* and upper GI endoscopy information simultaneously were included. The reasons for excluding 12,443 cases were as follows: (1) younger than 18 years, (2) repeated endoscopies or UBT test, (3) history of gastric surgery, (4) unavailable biopsy results, or (5) taking antibiotics, bismuth, proton pump inhibitors, and H2 receptor antagonists in the previous month. Finally, 69,071 subjects were enrolled, as presented in Figure 1. The Institutional Review Board of Tongji Hospital of Tongji Medical College (Wuhan, China) approved this study [TJ-IRB20230431]. Because we only analyzed de-identified data, the informed consent was waived.

### Endoscopic and histologic assessment

**Figure 2:**
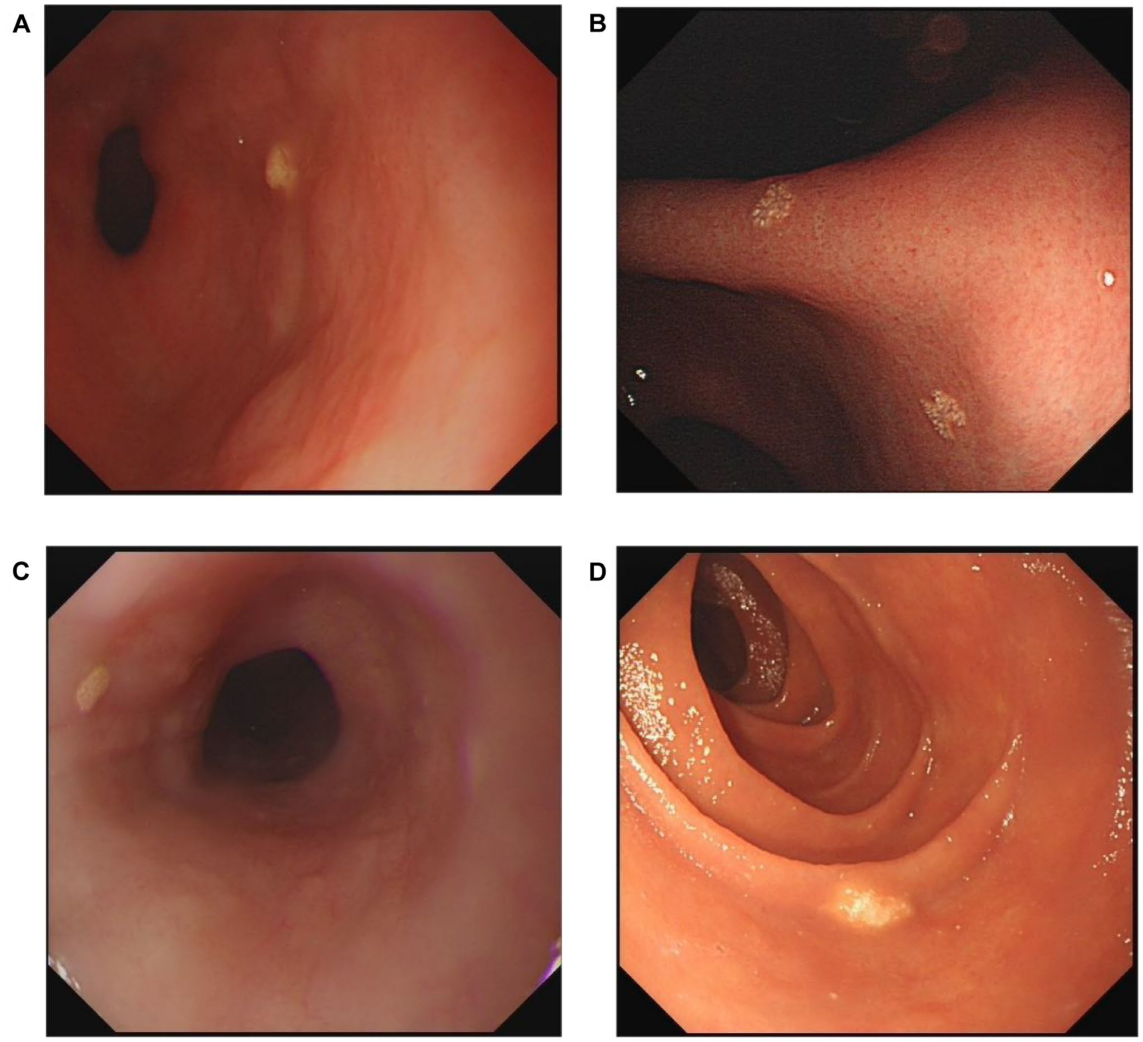
Representative endoscopic images of xanthelasma. **(A)** Single gastric xanthelasma. **(B)** Multiple gastric xanthelasmas. **(C)** Esophageal xanthelasma. **(D)** Duodenal xanthelasma.

Endoscopic diagnoses were recorded, including superficial gastritis, gastric polyp, bile reflux, peptic ulcer, reflux esophagitis, Barrett’s esophagus, and GX. As shown in Figure 2, xanthelasma was diagnosed by the appearance of yellow-white, slightly elevated or flat plaques. Single and multiple xanthelasmas were defined as having only one xanthelasma and more than two, respectively. Single-focal GX was determined as the GX distribution in one location (including gastric cardia, fundus, corpus, angle and antrum). At the same time, multifocal GXs meant the GX distribution in more than two locations. Any disagreement or uncertain diagnosis was resolved through further discussion with senior endoscopists. In addition, esophageal cancer, AG, IM, dysplasia, and gastric cancer were identified according to pathological findings. Histology was independently determined by two experienced pathologists, who were blinded to the results of the endoscopic examination.

### Statistical analysis

Statistical analyses were carried out using SPSS statistics version 23. Data for age was expressed as the mean ± standard error (SD), while other categorical variables were reported as frequency (%). Mann–Whitney U-test and Chi-squared test were used for between-group differences. Odds ratio (OR) and 95% confidence intervals (CI) were adopted to explore associations. OR with corresponding 95% CI were derived from logistic regression analysis. Two-sided *p* values less than 0.05 were considered to be significant.

## Results

### General characteristics of subjects with and without xanthelasma

A total of 69,071 cases were enrolled in the present study. The mean age was 45.8 ± 12.4 years, and 51.5% of the subjects were male. A summary of the characteristics divided into GX, esophageal/duodenal xanthelasma, and non-xanthelasma groups is shown in Table 1. Among all patients, 1,274 upper GI xanthelasmas (stomach:1220; esophagus:17; duodenum: 37) were diagnosed, with a prevalence of 1.84%.

**Table 1:** Baseline characteristics of the study population.

	All participants (*n* = 69,071)	Non-xanthelasma (*n* = 67,797)	Gastric xanthelasma (*n* = 1,220)	^a^*p* value	Esophageal xanthelasma (*n* = 17)	^b^*p* value	Duodenal xanthelasma (*n* = 37)	^c^*p* value
Age (years)	45.8 ± 12.4	45.7 ± 12.4	53.1 ± 9.5	<0.001	46.3 ± 12.8	0.874	48.2 ± 10.3	0.158
Male sex	35,589 (51.5%)	34,948 (51.5%)	615 (50.4%)	0.43	6 (35.3%)	0.180	20 (54.1%)	0.760
Superficial gastritis	21,477 (31.1%)	21,262 (31.4%)	201 (16.5%)	<0.001	2 (11.8%)	0.082	12 (32.4%)	0.888
Gastric polyp	6,073 (8.8%)	5,951 (8.8%)	115 (9.4%)	0.428	1 (5.9%)	1.000	6 (16.2%)	0.191
Polyp histopathology				0.636		0.424		0.122
Hyperplastic	1,594 (2.3%)	1,568 (2.3%)	26 (2.1%)		0 (0.0%)		0 (0.0%)	
Fundic gland	1,657 (2.4%)	1,623 (2.4%)	31 (2.5%)		1 (5.9%)		2 (5.4%)	
Pathological absence	2,822 (4.1%)	2,760 (4.1%)	58 (4.8%)		0 (0.0%)		4 (10.8%)	
Bile reflux	4,055 (5.9%)	3,986 (5.9%)	67 (5.5%)	0.568	0 (0.0%)	0.607	2 (5.4%)	1.000
Gastric/Duodenal ulcer	13,315 (19.3%)	13,060 (19.3%)	245 (20.1%)	0.473	4 (23.5%)	0.890	6 (16.2%)	0.638
Reflux esophagitis	5,524 (8.0%)	5,439 (8.0%)	82 (6.7%)	0.097	1 (5.9%)	1.000	2 (5.4%)	0.777
Barrett’s esophagus	3,074 (4.5%)	3,011 (4.4%)	62 (5.1%)	0.282	1 (5.9%)	1.000	0 (0.0%)	0.362
Esophageal cancer	160 (0.2%)	159 (0.2%)	1 (0.1%)	0.425	0 (0.0%)	1.000	0 (0.0%)	1.000
Gastric atrophy	10,203 (14.8%)	9,674 (14.3%)	523 (42.9%)	<0.001	3 (17.6%)	0.959	3 (8.1%)	0.284
Intestinal metaplasia	13,736 (19.9%)	13,091 (19.3%)	633 (51.9%)	<0.001	5 (29.4%)	0.455	7 (18.9%)	0.952
Dysplasia	494 (0.7%)	478 (0.7%)	16 (1.3%)	0.013	0 (0.0%)	1.000	0 (0.0%)	1.000
Gastric cancer	633 (0.9%)	614 (0.9%)	18 (1.5%)	0.038	1 (5.9%)	0.376	0 (0.0%)	1.000
*H. pylori* infection	18,750 (27.1%)	18,317 (27.0%)	422 (34.6%)	<0.001	5(29.4%)	1.000	6 (16.2%)	0.139

a Two-sided *p* values for the difference between gastric xanthelasma and non-xanthelasma.

b Two-sided *p* values for the difference between esophageal xanthelasma and non-xanthelasma.

c Two-sided p values for the difference between duodenal xanthelasma and non-xanthelasma. Age is expressed as mean (SD) and all other data are expressed as number (proportion). *H. pylori, Helicobacter pylori*.

The average ages of GX patients and patients without xanthelasma were 53.1 years and 45.7 years, respectively (*p* < 0.001). The percentages of superficial gastritis among patients with GX and without xanthelasma were 16.5 and 31.4%, respectively (*p* < 0.001). Moreover, the group with GX had a greater proportion of AG (42.9 vs. 14.3%, *p* < 0.001), IM (51.9 vs. 19.3%, *p* < 0.001), dysplasia (1.3 vs. 0.7%, *p* = 0.013), gastric cancer (1.5 vs. 0.9%, *p* = 0.038), and *H. pylori* infection (34.6 vs. 27.0%, *p* < 0.001) as compared to the non-xanthelasma group. However, no significant differences were found in sex, gastric polyp, bile reflux, gastric/duodenal ulcer, reflux esophagitis, Barrett’s esophagus, and esophageal cancer between the GX and non-xanthelasma groups. Additionally, there were no significant differences in all factors described in Table 1 between the esophageal/duodenal xanthelasma and the non-xanthelasma groups.

### Independent risk factors for GX

The multivariate logistic regression analysis encompassed indicators with *p* values less than 0.1 in Table 1. The results manifested that advanced age (OR = 1.040, 95%CI: 1.034–1.045), AG (OR = 1.833, 95%CI: 1.559–2.155), IM (OR = 2.415, 95%CI: 2.406–2.851), and *H. pylori* infection (OR = 1.324, 95%CI: 1.173–1.495) were independently related to GX (Table 2). In addition, we conducted a subgroup analysis to explore the relationship between the position of AG/IM and GX, and the results showed no correlation between them (Table 3).

**Table 2:** Multivariate analysis for the risk factors of gastric xanthelasma.

	Crude OR (95%CI)	*p* value	Adjusted OR (95%CI)	*p* value
Age (years)	1.052 (1.047–1.057)	<0.001	1.040 (1.034–1.045)	<0.001
Superficial gastritis	0.432 (0.371–0.503)	<0.001	1.040 (0.874–1.239)	0.657
Reflux esophagitis	0.826 (0.659–1.035)	0.097	0.827 (0.659–1.039)	0.103
Gastric atrophy	4.508 (4.017–5.060)	<0.001	1.833 (1.559–2.155)	<0.001
Intestinal metaplasia	4.506 (4.021–5.050)	<0.001	2.415 (2.406–2.851)	<0.001
Dysplasia	1.872 (1.134–3.090)	0.014	1.161 (0.698–1.931)	0.565
Gastric cancer	1.639 (1.022–2.627)	0.040	1.232 (0.761–1.996)	0.396
*H. pylori* infection	1.429 (1.268–1.609)	<0.001	1.324 (1.173–1.495)	<0.001

OR, odds ratio; CI, confidence intervals.

**Table 3:** Location characteristics of AG/IM with or without GX.

Location	AG without GX (*n* = 9,674)	AG with GX (*n* = 523)	*p* value
Cardia/Fundus	44 (0.5%)	3 (0.6%)	0.953
Corpus	120 (1.2%)	7 (1.3%)	0.844
Angle/Antrum	5,728 (59.2%)	312 (59.7%)	0.840
Multifocal	3,782 (39.1%)	201 (38.4%)	0.762
Location	IM without GX (*n* = 13,091)	IM without GX (*n* = 633)	*p* value
Cardia/Fundus	178 (1.4%)	6 (0.9%)	0.379
Corpus	188 (1.4%)	8 (1.3%)	0.721
Angle/Antrum	8,297 (63.4%)	382 (60.3%)	0.122
Multifocal	4,428 (33.8)	237 (37.4%)	0.061

AG, atrophic gastritis; IM, intestinal metaplasia; GX, Gastric xanthelasma.

### Association between *Helicobacter pylori* infection, IM or AG, and GX

Based on the different statuses of *H. pylori* and AG, the subjects were divided into four groups: *H. pylori* (−) & AG (−), *H. pylori* (−) & AG (+), *H. pylori* (+) & AG (−), and *H. pylori* (+) & AG (+). As shown in Table 4, compared to the *H. pylori* (−) & AG (−) group, the *H. pylori* (+) & AG (−), *H. pylori* (−) & AG (+), and *H. pylori* (+) & AG (+) groups had approximately 1.6-fold risk, 3.6-fold risk and 4.0-fold risk for GX, respectively.

**Table 4:** Association between IM or AG, *H. pylori* infection, and gastric xanthelasma.

Groups	Gastric xanthelasma	Non-xanthelasma	Crude OR (95%CI)	*p* value	^a^Adjusted OR (95%CI)	*p* value
*H. pylori* (−) & AG (−)	461 (1.06%)	43,172	1		1	
*H. pylori* (+) & AG (−)	236 (1.55%)	14,951	1.478 (1.262–1.731)	<0.001	1.563 (1.331–1.836)	<0.001
*H. pylori* (−) & AG (+)	337 (5.07%)	6,308	5.003 (4.337–5.772)	<0.001	3.637 (3.119–4.241)	<0.001
*H. pylori* (+) & AG (+)	186 (5.24%)	3,366	5.175 (4.349–6.157)	<0.001	4.016 (3.340–4.828)	<0.001
*H. pylori* (−) & IM (−)	381 (0.93%)	40,488	1		1	
*H. pylori* (+) & IM (−)	206 (1.43%)	14,218	1.540 (1.298–1.826)	<0.001	1.637 (1.376–1.947)	<0.001
*H. pylori* (−) & IM (+)	417 (4.43%)	8,992	4.928 (4.281–5.673)	<0.001	3.885 (3.327–4.536)	<0.001
*H. pylori* (+) & IM (+)	216 (5.01%)	4,099	5.600 (4.724–6.638)	<0.001	4.552 (3.787–5.471)	<0.001

a Adjusted for age, sex, superficial gastritis, gastric polyp, bile reflux, gastric/duodenal ulcer, reflux esophagitis, Barrett’s esophagus, esophageal cancer, dysplasia, and gastric cancer by logistic regression analysis. AG, atrophic gastritis; IM, intestinal metaplasia; *H. pylori, Helicobacter pylori*.

Based on the different statuses of *H. pylori* and IM, the subjects were classified into four groups: *H. pylori* (−) & IM (−), *H. pylori* (−) & IM (+), *H. pylori* (+) & IM (−), and *H. pylori* (+) & IM (+). As reported in Table 4, The prevalence of GX in the *H. pylori* (+) & IM (+) group was the highest among the four groups (5.01%). Compared to the *H. pylori* (−) & IM (−) group, the *H. pylori* (+) & IM (−), *H. pylori* (−) & IM (+), and *H. pylori* (+) & IM (+) groups had approximately 1.6-fold risk, 3.9-fold risk and 4.6-fold risk for GX, respectively.

### Association between the number of GX and histopathologic findings

Subgroup analysis compared histopathologic findings between subjects with single and multiple GXs. We found that the incidence of AG and IM in patients with multiple GXs was significantly higher than in patients with single GX. However, there was no significant correlation between the number of GX and dysplasia, gastric cancer, or *H. pylori* positivity. Also, patients with multiple GXs were further divided into two groups according to having either single-focal or multifocal GXs. No significant relationship was found between the GX distribution and histopathologic findings (Table 5).

**Table 5:** Comparison of histopathologic findings according to the number, and number of localizations of gastric xanthelasmas.

	Single xanthelasma (*n* = 920)	Multiple xanthelasmas (*n* = 300)	*p* value
Gastric atrophy	366 (39.8%)	157 (52.3%)	<0.001
Intestinal metaplasia	455 (49.5%)	178 (59.3%)	0.003
Dysplasia	9 (1.0%)	7 (2.3%)	0.134
Gastric cancer	11 (1.2%)	7 (2.3%)	0.253
*H. pylori* infection	318 (34.6%)	104 (34.7%)	0.974
	Single-focal xanthelasma (*n* = 202)	Multifocal xanthelasmas (*n* = 98)	*p* value
Gastric atrophy	101 (50.0%)	42 (42.9%)	0.245
Intestinal metaplasia	120 (59.4%)	58 (59.2%)	0.971
Dysplasia	5 (2.5%)	2 (2.0%)	1.000
Gastric cancer	4 (2.0%)	3 (3.1%)	0.862
*H. pylori* infection	74 (36.6)	30 (30.6%)	0.304

*H. pylori*, *Helicobacter pylori*.

## Discussion

The present analysis supported that *H. pylori* infection, AG, and IM were independent risk factors of GX. It also revealed that the GX risk might increase further in individuals with *H. pylori*-related AG/IM. As for esophageal/duodenal xanthelasma, the connection between it and upper GI diseases was not discovered.

Due to the rarity of esophageal/duodenal xanthelasma, there have been few reports on it. Gencosmanoglu (7) showed that the incidence of xanthelasma in the stomach, esophagus, and duodenum was 76, 12, and 12%, respectively. Although the etiology of xanthelasma is still unknown, most authors believed xanthelasma might be related to inflammation caused by mucosal damage or aging (20, 21). This may explain why xanthelasma incidence in the esophagus was lower than in the stomach, as the esophageal mucosa has better damage tolerance. (7). To date, no research has explored whether esophageal/duodenal xanthelasma has the same significance as GX. Our study found that unlike GX, esophageal/duodenal xanthelasma may not be associated with upper GI diseases, or more specifically, *H.* pylori infection, AG, IM, and gastric cancer. However, this discovery requires further confirmation from larger sample size studies, and the etiology and specific mechanism still need to be investigated.

It is well known that *H. pylori* can cause various GI diseases, such as peptic ulcer, AG, IM, and gastric cancer (22). Prior small-sample studies also showed a positive correlation between *H. pylori* and GX (16, 23). However, other studies did not find a relation between *H. pylori* and GX (13, 17, 18). This contradiction between studies might be partly due to different rates of *H. pylori* infection in different regions, small sample sizes, and various detection methods. In 1996, Hori (16) conducted *H. pylori* antigen localization analysis on 145 xanthelasma biopsy specimens and identified *H. pylori* infection on the surface of concave cells in 69 (48%) samples. More than half of the antigens were penetrated into the lamina propria. These findings may support the hypothesis that *H. pylori* infection is the etiology of some xanthelasmas.

Patients with AG/IM raise the risk of gastric adenocarcinoma. For advanced AG or extensive IM, an endoscopic examination every three years is recommended (24). Multiple publications have noted a positive relationship between GX and AG/IM (8, 13, 19, 25). The current study also demonstrated that AG and IM were independent risk factors for GX. However, no relationship was found between the location distribution of AG/IM and GX. To the best of our knowledge, no research has clarified the association between GX and *H. pylori*-related AG/IM. Therefore, we conducted a further analysis that stratified cases based on *H. pylori* infection and AG/IM statuses. The results indicated that patients with simple *H. pylori* infection had a 1.6-fold risk for GX, which increased when *H. pylori* infection caused AG/IM (OR 4.0–4.6). In addition, our study found that multiple GXs had a higher rate of IG/IM than single GX, which was consistent with the findings of Köksal (8). Our result may indicate that the higher the number of GX, the greater the likelihood of IG/IM.

Moreover, Sekikawa (9, 10) and Shibukawa (26, 27) believed that the presence of GX was a predictive indicator of gastric cancer. Kaiserling (28) speculated that the increased release of oxygen free radicals might simultaneously play a role in the development of gastric cancer and GX. In this analysis, although the Chi-square test indicated that gastric cancer was more common in individuals with GX (1.5 vs. 0.9%, *p* = 0.038), multivariate analysis did not find an independent correlation between gastric cancer and GX, which might be related to a lower detection rate of gastric cancer in our cohort.

Several limitations of our study must be considered. Firstly, a retrospective single-center study design increased the possibility of bias. Secondly, information on the history of *H. pylori* eradication was not available. Thirdly, we did not evaluate interobserver variability in assessing endoscopic findings. Furthermore, we could not obtain the specific stages of the Operative Link on Gastritis Assessment (OLGA) and Operative Link on Gastritis/Intestinal-Metaplasia Assessment (OLGIM) due to insufficient gastric biopsy sites. Despite these shortcomings, our study elucidated the relationship between GX and upper GI diseases with a relatively large sample size to determine whether xanthelasma could serve as a warning sign for gastric precancerous lesions. Moreover, this is the first report on risk factors for esophageal/duodenal xanthelasma.

In summary, the results demonstrated an increased risk of GX in subjects with *H. pylori* infection, AG and IM, especially *H. pylori*-related AG/IM. Although GX is a benign lesion, sufficient attention should be given during the endoscopic examination. Endoscopists should spend more time on patients with gastric xanthelasma, especially those with multiple GXs, to rule out *H. pylori* infection, precancerous lesions, and even gastric cancer.

## Data availability statement

The original contributions presented in the study are included in the article/supplementary material, further inquiries can be directed to the corresponding author.

## Ethics statement

The studies involving humans were approved by this study has been approved by the Ethical Committees of Tongji Hospital of Tongji Medical College [TJ-IRB20230431]. The studies were conducted in accordance with the local legislation and institutional requirements. The ethics committee/institutional review board waived the requirement of written informed consent for participation from the participants or the participants’ legal guardians/next of kin because the informed consent was waived as only de-identified data was analyzed.

## Author contributions

LF, JG, YZ, RD, and YH conceived and designed this study. JG, YZ, MZ, LF, SX, and JL participated in data collection, interpretation and analysis. LF, MZ, YH, KZ, and FX wrote the first draft of the manuscript. LF, JG, RD, KZ, and SX prepared the figures and tables. JL and FX revised the primary manuscript. All authors contributed to the article and approved the submitted version.

## Funding

This work is supported by the Subproject of the National Key Research and Development Program (no. 2021YFC2600263).

## Conflict of interest

The authors declare that the research was conducted in the absence of any commercial or financial relationships that could be construed as a potential conflict of interest.

## Publisher’s note

All claims expressed in this article are solely those of the authors and do not necessarily represent those of their affiliated organizations, or those of the publisher, the editors and the reviewers. Any product that may be evaluated in this article, or claim that may be made by its manufacturer, is not guaranteed or endorsed by the publisher.
